# NCBITaxonomy.jl: rapid biological names finding and reconciliation

**DOI:** 10.1186/s12862-025-02425-4

**Published:** 2025-08-20

**Authors:** Timothée Poisot, Rory Gibb, Sadie J. Ryan, Colin J. Carlson

**Affiliations:** 1 Départment de Sciences Biologiques, Université de Montréal, Montréal, QC Canada; 2 Québec Centre for Biodiversity Science, Montréal, QC Canada; 3 Centre on Climate Change and Planetary Health, London School of Hygiene and Tropical Medicine, London, UK; 4 Centre for Mathematical Modelling of Infectious Diseases, London School of Hygiene and Tropical Medicine, London, UK; 5 Centre for Biodiversity and Environment Research, University College London, London, UK; 6 Emerging Pathogens Institute, University of Florida, Gainesville, FL USA; 7 School of Life Sciences, University of KwaZulu-Natal, Durban, South Africa; 8 Department of Geography, University of Florida, Gainesville, FL USA; 9 Department of Epidemiology of Microbial Diseases, Yale School of Public Health, New Haven, CT USA

**Keywords:** NCBI, Taxonomic names, ICTV, Database

## Abstract

NCBITaxonomy.jl is a Julia package designed to address the complex challenges of taxonomic name reconciliation using a local copy of the NCBI taxonomic backbone (Federhen in Nucleic Acids Res 40:D136–D143, 2012, Schoch et al. in Database 2020:baaa062, 2020). The package provides advanced name matching capabilities that handle common issues in taxonomic data, including synonyms, homonyms, vernacular names, nomenclatural changes, and typographical errors. Core functionalities include case-insensitive search, customizable fuzzy string matching, and taxonomically-restricted searches. The package implements a robust exception system that explicitly handles ambiguous matches without interrupting workflow execution, enabling automated processing of large datasets. NCBITaxonomy.jl works with Julia 1.6 and up, uses Apache Arrow format for efficient local storage. It provides lineage navigation and taxonomic distance functions. The package has been successfully deployed in large-scale projects for automated name reconciliation and cleaning, demonstrating its effectiveness for high-throughput name reconciliation across heterogeneous biological datasets. The design prioritizes programmatic access over command-line usage, making it well-suited for integration into bioinformatics pipelines requiring reliable taxonomic standardization.

## Background

Unambiguously identifying species names in text is a far more challenging task than it may appear. There are a vast number of reasons for this. Different databases keep different taxonomic “backbones”, *i.e.* different data structures in which names are mapped to species, and organised in a hierarchy. Not all names are unique identifiers to groups. For example, *Io* can either refer to a genus of plants from the aster family, or to a genus of molluscs; the genus *Mus* (of which the house mouse *Mus musculus* is a species), contains a sub-genus *also* named *Mus* (within which *Mus musculus* is located). Conversely, the same species can have several names, which are valid synonyms: for example, the domestic cow *Bos taurus* admits *Bos primigenius taurus* as a valid synonym. In addition to binomial names, the same species can be known by many vernacular (common) names, which are language or even region-specific: *Ovis aries*, for example, has valid English vernaculars including lamb, sheep, wild sheep, and domestic sheep.

In addition, taxonomic nomenclature changes regularly, with groups being split, merged, or moved to a new position in the tree of life; often, taxonomic revisions lead to these events occurring simultaneously. This is, notably, a common occurrence with viral taxonomy, each subsequent version of which can differ markedly from the last; compare, *e.g.* [10–17], where entire viral sub-trees were split, re-organized, and created within just two years. As a consequence any mapping of names to other biological entities can become outdated, and therefore invalid. These taxonomic changes have profound implications for the way we perceive biodiversity at global scales [7], to the point where taxonomic revisions should sometimes be actively conducted to improve *e.g.* conservation outcomes [11].

None of these issues, were they to happen in isolation, would be very difficult to deal with. Indeed, performing the lookup for any text string in any database is a trivial operation. But to add to the complexity, one must also consider that most taxa names are at some point manually typed, which has the potential to introduce additional sources of variation in raw data; it is likely to expect that such mistakes may arise when attempting to write down the (perfectly valid) names of the bacterial isolate known as *Myxococcus llanfairpwllgwyngyllgogerychwyrndrobwllllantysiliogogogochensis*, or of the crowned slaty flycatcher *Griseotyrannus aurantioatrocristatus*. These mistakes are more likely when dealing with hyper-diverse samples (demanding to memorize more names), like plant census [5, 6, 16], when dealing with multiple investigators with different knowledge of the taxonomy; and as a result of the estimated error in any data entry exercise, which other fields estimate at up to about 5% [1]. As a result, the first question one needs to ask when confronted with a string of characters that purportedly points to a node in the tree of life is not “to which entry in the taxonomy database is it associated?”, but “is there a mistake in this name that is likely to render a simple lookup invalid?”.

All these considerations become important when matching species names both within and across datasets. Let us consider the hypothetical species survey of riverine fishes: European chub, *Cyprinus cephalus*, *Leuciscus cephalus*, *Squalius cephalus*. All are the same species (*S. cephalus*), referred to as one of the vernacular (European chub) and two formerly accepted names now classified as synonyms (but still present in the literature). A simple estimate of diversity based on the user-supplied names would give $n=4$ species, when there is in fact only one. Some cases can be more difficult to catch; for example, the species *Isoetes minima* is frequently mentioned as *Isœtes minima*, because text processing use the “œ” grapheme to mark the “oe” diphthong. When the size of biodiversity datasets increases, and notably when the taxonomic scope of these datasets explodes, including organisms for which “names” are a fuzzier concept (for example, *Influenza A virus (A/Sydney/05/97-like(H3N2))* is a valid name for a common influenza strain, although one that lacks a taxonomic rank), the feasibility of manual curation decreases.

In this manuscript, we describe NCBITaxonomy.jl, a Julia package that provides advanced name matching and error handling capacities for the reconciliation of taxonomic names to the NCBI database. This package works by downloading a local copy of the taxonomy database, so that queries can be made rapidly, and that subsequent queries will return the same results. The package offers functionalities to automatically prompt users to update the local copy of the taxonomy database if it becomes outdated. This package was used to facilitate the development of the *CLOVER* [9] database of host-virus associations, by reconciling the names of viruses and mammals from four different sources, where all of the issues described above were present. More recently, it has become part of the automated curation of data for the *VIRION* [4] database, which automatically curates an up-to-date, authoritative virome network from dozens of heterogeneous sources. We describe the core capacities of this package, and highlight how it enables safe, high-performance name reconciliation.

## Implementation

Based on the author’s experience reconciling lists of thousands of biological names, NCBITaxonomy.jl is built around a series of features that allow (1) maximum flexibility when handling names without a direct match, (2) a bespoke exception system to handle failures to match automatically, and (3) limits to the pool of potential names in order to achieve orders-of-magnitude speedups when the broad classification of the name to match is known. Adhering to these design principles led to a number of choices. A comparison of the features of different packages, as inferred from their public documentation, is presented in Table 1.

**Table 1:** Comparison of core features of packages offering access to the NCBI taxonomic backbone

Tool	Lang	Library	CLI	Local DB	Fuzzy	Case	Subsets	Ranks	References
NCBITaxonomy.jl	Julia	✓		✓	✓	✓	✓	✓	This paper
taxadb	R	✓		✓			✓	✓	[12]
taxopy	Python	✓		✓		✓			[3]
rentrez	R	✓						✓	[18]
TaxonKit	Python		✓	✓					[14]
NCBI-taxonomist	Python		✓	✓					[2]

Library, ability to be called from code; CLI, ability to work as a command-line tool; Local DB, ability to store a copy of the database locally; Fuzzy, ability to perform fuzzy matching on inputs; Case, ability to perform case-insensitive search; Subsets, ability to limit the search to a subset of the raw database; Ranks, ability to limit the search to specific taxonomic ranks.

The features of the various packages have been determined from reading their documentation.

First, we specifically target programmatic (as opposed to command-line) based approaches, so that the functionalities of the package can be accessed as part of a larger pipeline. Second, to speed up the queries, we work from a local version of the database, the installation of which is handled at build time by the package itself; each project using the package can use its own version of the taxonomy by specifying a folder where it is stored through an environmental variable. Third, because we *cannot* trust that the names as presented in the original data are correct, we offer case-insensitive search (at no time cost) and fuzzy-matching (at a significant time cost). Either of these strategies can be called only after a case-sensitive, non-fuzzy search yields an exception about the lack of a direct match. Finally, in order to achieve a good performance even when relying on fuzzy matching, we offer the ability to limit the search to specific parts of the taxonomy database. An example of the impact of this feature on the performance of the package is presented in Table 1.

An up-to-date version of the documentation for NCBITaxonomy.jl can be found in the package’s *GitHub* repository (PoisotLab/NCBITaxonomy.jl), including examples and in-line documentation of every method. The package is released under the MIT license. Contributions can be made in the form of issues (bug reports, questions, features suggestions) and pull requests, all of which can be consulted publicly. Alternatively, the package can be downloaded from its Zenodo page (ID 7698661, along with a versioned DOI.

### Local file storage

In order to achieve good performance, the package will first retrieve the latest (as validated by its checksum) NCBI taxonomy database, store it locally, and pre-process it as a set of Julia data tables. By default, the taxonomy will be downloaded to the user’s home directory, which is not an ideal solution, and therefore we recommend that users set an environment variable to specificy where the data will be loaded from (this path will be created if it doesn’t exist):

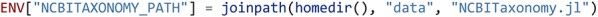



Note that this location can be different for different projects, as the package is able to update the taxonomic backbone (and will indeed prompt the user to do so if the taxonomy is more than 90 days old, as infered from looking at the raw files creation timestamp). The package can then be checked out and installed anonymously from the central Julia repository:

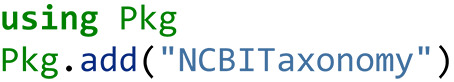



As long as the package is not re-built, the local set of tables downloaded from NCBI will not change; this way, users can re-run an analysis with a guarantee that the underlying taxonomic backbone has not changed, which is not the case when relying on API queries. In order to update the taxonomic backbone, users can call the build function of Julia’s package manager (]buildNCBITaxonomy), which will download the most recent version of all files.

This software note describes version v0.3.0 of the package (we follow semantic versioning), which works on Julia 1.5 upwards. The dependencies are all resolved by the package manager at installation, and (on the user-facing side) include the StringDistances.jl package, allowing users to experiment with different string matching methods. As is best practices for Julia packages, a Project.toml file specifying compatible dependencies versions is distributed with the package. The code is covered by unit-tests (with about 98% coverage), as well as integration tests as part of the documentation (specifically, a use-case detailing how to clean data from a biodiversity survey, and a use-case aiming to reconstruct a taxonomic tree for the Lemuriformes).

### Improved name matching

Name finding, *i.e.* the matching of an arbitrary string to a taxonomic identifier, is primarily done through the taxon function, which admits either a unique NCBI identifier (*e.g.* taxon(36,219) for the bogue *Boops boops*), a string (taxon("Boops boops")), or a data frame with a restricted list of names in order to create a name finder function (see the next section). The taxon method has additional arguments to perform fuzzy matching in order to catch possible typos (taxon("Boops bops"; strict = false)), to perform a lowercase search (useful when alphanumeric codes are part of the taxon name, like for some viruses), and to restrict the search to a specific taxonomic rank. The taxon function also accepts a preferscientificname keyword, to prevent matching vernacular names; the use of this keyword ought to be informed by knowledge about how the data were entered.

The lowercase search can be a preferable alternative to fuzzy string matching. Consider the string Adeno-associated virus 3b - it has three names with equal distance (under the Levensthein string distance function):


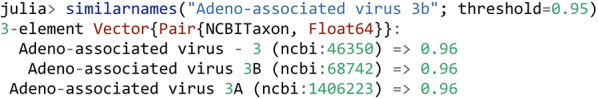


Depending on the operating system (and specifically whether it is case-sensitive), either of these three names can be returned; compare to the output of a case insensitive name search:

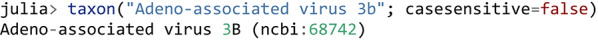



This returns the correct name.

### Name matching output and error handling

When it succeeds, taxon will return a NCBITaxon object (made of a name string field, and an id numerical field). That being said, the package is designed under the assumption that ambiguities should yield an error for the user to handle. There are two such errors: NameHasNoDirectMatch (with instructions about how to possibly solve it, using the similarnames function), or a NameHasMultipleMatches (listing the possible valid matches, and suggesting to use alternativetaxa to find the correct one). Therefore, the common way to work with the taxon function would be to wrap it in a try/catch statement:


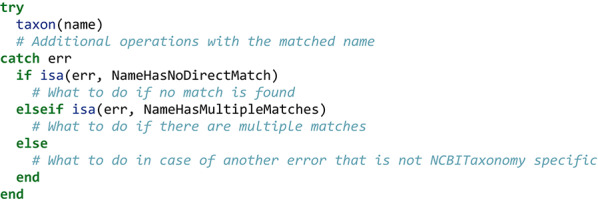


These functions will not demand any user input in the form of key presses (though they can be wrapped in additional code to allow it), as they are intended to run on clusters or virtual machines without supervision. The taxon function has good scaling using muliple threads. For convenience in rapidly getting a taxon for demonstration purposes, we also provide a string macro, whereby *e.g.* ncbi"Procyon lotor" will return the taxon object for the raccoon.

### Name filtering functions

As the full NCBI names table holds over 3 million entries at the time of writing, we have provided a number of functions to restrict the scope of names that are searched. These are driven by the NCBI *divisions*. For example nf = mammalfilter(true) will return a data frame containing the names of mammals, inclusive of rodents and primates, and can be used with *e.g.* taxon(nf,"Pan"). This has the dual advantage of making queries faster, but also of avoiding matching on names that are shared by another taxonomic group (which is not an issue with *Pan*, but is an issue with *e.g. Io* as mentioned in the introduction, or with the common name *Lizard*, which fuzzy-matches on the hemipteran genus *Lisarda* rather than the class *Lepidosauria*).

Note that the use of a restricted list of names can have significant performance consequences. This is illustrated in Table 2. When possible, the optimal search strategy is to (i) rely on name filters to ensure that searches are conducted within the appropriate NCBI division, and (ii) only rely on fuzzy matching when the strict or lowercase match fails to return a name, as fuzzy matching can result in order of magnitude more run time and memory footprint.

**Table 2:** Time and performance of different search strategies for the string "chimpanzees"

Names list	Fuzzy matching	Time (ms)	Allocations	Memory footprint
all	no	23	34	2 KiB
	yes	105	2580	25 MiB
mammalfilter(true)	no	0.55	32	2 KiB
	yes	1.9	551	286 KiB
primatefilter(true)	no	0.15	33	2 KiB
	yes	0.3	92	27 KiB

These numbers were obtained on a single Intel i7-8665U CPU (1.90GHz). Using "Pan" as the search string (for which "chimpanzees"is a recognized vernacular) gave qualitatively similar results, suggesting that there is no performance cost associated with working with synonyms or vernacular input data

### Quality of life functions

In order to facilitate working with names, we provide the authority function (gives the full taxonomic authority for a name), synonyms (to get alternative valid names), vernacular (for English common names), and rank (for the taxonomic rank). These functions are not used in name matching, but are often useful in the post-processing of results.

### Taxonomic lineages navigation

The children function will return all nodes that are directly descended from a taxon; the descendants function will recursively apply this function to all descendants of these nodes, until only terminal leaves are reached. The parent function is an “upwards” equivalent, giving the taxon from which a taxon descends; the lineage function chains calls to parent until either taxon(1) (the taxonomy root) or an arbitrary ancestor is reached.

The taxonomicdistance function (and its in-place equivalent, taxonomicdistance!, which uses memory-efficient re-allocation if the user needs to change the distance between taxonomic ranks) uses the [15] approach to reconstruct a matrix of distances based on taxonomy, which can serve as a rough proxy when no phylogenies are available. This allows coarse estimations of taxonomic diversity based on species lists. The default distance between taxonomic levels is as in [15] (*i.e.* species have a distance of 0, genus of 1, family of 2, sub-classes of 3, and everything else 4), but specific scores can be passed for *any* taxonomic level know to the NCBI name table.

## Conclusion

NCBITaxonomy.jl enables rapid, taxonomically-restricted, adaptive matching for taxonomic names. By implementing various combinations of search strategies, it allows users to (i) optimize the speed of their queries and (ii) avoid usual caveats of simple string matching. Through explicit exceptions, it allows to write code that will handle the possible edge cases that cannot be solved automatically in a way that does not interrupt execution, or requires manual input by the user. Given the breadth of the NCBI taxonomy database, NCBITaxonomy.jl is particularly suited to the name cleaning of large datasets of names.

## Data Availability

Not applicable.
